# Microglia in Retinal Degeneration

**DOI:** 10.3389/fimmu.2019.01975

**Published:** 2019-08-20

**Authors:** Khalid Rashid, Isha Akhtar-Schaefer, Thomas Langmann

**Affiliations:** ^1^Laboratory for Experimental Immunology of the Eye, Department of Ophthalmology, Faculty of Medicine and University Hospital Cologne, University of Cologne, Cologne, Germany; ^2^Center for Molecular Medicine Cologne, Cologne, Germany

**Keywords:** retina, microglia, neuroprotection, chronic inflammation, immunomodulation

## Abstract

The retina is a complex tissue with multiple cell layers that are highly ordered. Its sophisticated structure makes it especially sensitive to external or internal perturbations that exceed the homeostatic range. This necessitates the continuous surveillance of the retina for the detection of noxious stimuli. This task is mainly performed by microglia cells, the resident tissue macrophages which confer neuroprotection against transient pathophysiological insults. However, under sustained pathological stimuli, microglial inflammatory responses become dysregulated, often worsening disease pathology. In this review, we provide an overview of recent studies that depict microglial responses in diverse retinal pathologies that have degeneration and chronic immune reactions as key pathophysiological components. We also discuss innovative immunomodulatory therapy strategies that dampen the detrimental immunological responses to improve disease outcome.

## Introduction

The retina is part of the central nervous system with more than 60 distinct retinal cell types, high level organization, and a evolutionarily conserved structure (1, 2). The mammalian retina contains three distinct glia cells types; Müller cells, astrocytes, and microglia (3, 4). Müller cells are specialized radial cells that span all retinal layers and account for about 90% of the retinal glia population (3, 5). They support functioning and metabolism of retinal neurons by releasing trophic factors, recycling neurotransmitter glutamate, and controlling extracellular ion homeostasis (6). Retinal astrocytes perform similar functions to Müller cells, ranging from neurotrophic, metabolic and mechanical support of neurons, and maintenance of the blood retinal barrier (3, 7). Retinal astrocytes are however almost entirely restricted to the nerve fiber layer, and to a lesser extent to the ganglion cell layer (7, 8). Microglia, the third glial cell type, represent the resident tissue macrophages and play important roles in retinal homeostasis, recovery from injury and progression of disease (9, 10).

As early as 1897, Spanish neuroscientist Ramón y Cajal began to appreciate that there were more cell types in the brain than just neurons and astrocytes (11, 12). In pursuit of this hypothesis, he developed the gold chloride sublimate staining approach that labeled astrocytes and other cells that he named the “third element” (12, 13). Del Río-Hortega, another Spanish neuroscientist later separated the cells in the “third element,” becoming the first scientist to describe microglia as distinct cellular entity (12, 14). He described their ramified morphology in the normal brain using elegant drawings and provided evidence of their morphological transformations during tissue brain pathology (12, 14). These tremendous findings have withstood the test of time and remain highly relevant today.

In the normal retina, microglia cells are located in the plexiform layers where they exhibit elaborate ramified processes responsible for immune surveillance of the retina (4). Noxious insults in the retina such as oxidative stress, hypoxia or inherited mutations trigger microglia reactivity manifested by amoeboid morphology, increased proliferation and migration to the sites of injury (10, 15). While this initial “constructive” inflammatory response can rapidly enhance tissue repair and return to homeostasis, sustained microglial inflammatory responses can instigate severe alterations in retinal integrity, and aggravate neuronal demise (16). This review therefore offers a comprehensive overview on the dual role of microglia in the retina, examining their contribution in various retinal neurodegenerative disorders and in aging and parainflammation. In addition, we discuss recent strategies that have been shown to suppress microglial inflammatory responses in the retina and prevent neuronal cell death.

## Origin and Maintenance of Microglia in the Retina

Earlier attempts to find answers to the uncertainties existing around the origin and maintenance of retinal microglia were made by transplanting bone marrow (BM) derived cells from eGFP-transgenic mice into irradiated normal adult mice (17). Retinal flat mounts of the recipient mice were found to contain BM derived eGFP^+^ cells as early as 8-weeks post transplantation, and by the 6th month, all retinal myeloid cells were eGFP^+^ (17). Using *CX3CR1-GFP/*+ mice as BM donors, where GFP expression is restricted to monocyte lineage cells, a separate study observed recruitment of monocyte-derived cells into the retinal tissue 4 weeks post-transplantation (18). Of note, both studies observed that under steady state conditions, retinal microglia exhibited very low proliferation rates (17, 18). However, in a separate study where mice were irradiated and injected with eGFP+ BM cells prior to induction of retinal injury, only a minute number of donor cells were observed in the uninjured normal retina up to 12 months following BM transplantation (19). In contrast, a massive recruitment of BM derived cells into the retina was observed following injury (19). The authors partially attributed the low recruitment of donor cells to the uninjured eye to shielding eyes and heads of mice prior to irradiation (19). They argued that in the previous experiments, irradiation could have unintentionally damaged photoreceptors and enhanced migration of BM-derived cells to the injured retina (19). Indeed, additional factors not mentioned by the authors, such as large amounts of circulating cytokines, CNS vascular changes, and temporary disruptions of the blood-brain barrier alterations may confound interpretation of results following irradiation (20, 21).

Therefore, to further analyse the contribution of circulating BM derived progenitors on the turnover of resident microglia in the CNS, a separate study employed parabiosis experiments (22). Parabiosis, the surgical joining of two organisms, allowed for the sharing of the blood circulation between wild type and GFP transgenic mice without affecting their blood-brain nor the blood-retinal barrier (21–23). The obtained results revealed that under steady-state conditions, CNS microglia were a closed system with the capacity to self-renew and were not replenished by BM derived cells (22). Indeed, it was later established that microglia were derived from primitive myeloid progenitors originating from the yolk sac and that postnatal hematopoietic progenitors did not significantly contribute to microglia homeostasis (24).

A subsequent study (25) demonstrated that the brain harbored latent nestin^+^ microglial progenitors throughout the CNS (25). Pharmacological depletion of microglia using selective CSF1R inhibitor PLX3397 triggered rapid proliferation of nestin^+^ cells throughout the CNS, which later differentiated into microglia and repopulated the entire brain within 1 week of inhibitor cessation (25). Interestingly, this regeneration mirrored some aspects of normal development, as embryonic stem cells require a nestin^+^ stage on their way to becoming microglia (25, 26). However, subsequent microglia ablation studies have strongly disputed the presence of nestin^+^ microglia progenitor cells and instead provide evidence that microglia repopulation following pharmacological or genetic depletion occurs solely from residual non-depleted internal pools which exert massive proliferation and transiently express nestin (27–29). Similarly, retinas depleted of microglia using selective CSF1R inhibitor PLX5622 were not repopulated from nestin^+^ precursors (30, 31). Rather, repopulated retinal microglia exhibited dual extra retinal origins; first from residual microglia in the optic nerve and secondly from ciliary body/iris macrophages (30). Residual optic nerve microglia repopulated the retina along the center-to-periphery axis, while macrophages in the ciliary body/iris repopulated the retina along the periphery and accounted for around 15% of the repopulated microglia (30). Of note, these findings uncovered for the first time radial migratory routes of retinal microglia and demonstrated the presence of peripheral macrophage-derived microglia which were significantly less ramified than their central counterparts (30). In a parallel study, it was shown that microglia repopulation in the retina was regulated via neuronal-microglial crosstalk in the form of CX3CL1-CX3CR1 signaling which potentiated microglia proliferation and morphological maturation (31). Most importantly, the latter study demonstrated that that the repopulated cells fully restored microglia functions in the retina including immunosurveillance and synaptic maintenance (31).

## Microglia in the Healthy Retina

Microglial cells play active roles in maintaining the normal structure and functioning of the retina. During retinal development, microglia cells are largely confined to the ganglion cell layer and inner plexiform layer, where they phagocytose cellular corpses of excessively produced retinal ganglion cells (RGCs) early in development (32). Moreover, in the early postnatal stages when there is a robust synaptic remodeling, microglia are involved in pruning of weak presynaptic terminals of RGCs (33). This process occurs in a complement C3-CR3-dependent mechanism, where activated C3 (iC3b/C3b) selectively labels the weak RGCs terminals triggering a C3-receptor dependent phagocytosis pathway in microglia (4, 33). Importantly, microglial dependent apoptosis of RGCs and the elimination of costly neural connections deemed unfit for proper functioning plays a crucial role in the normal postnatal development of the retina and cortical visual areas (32, 33).

Following the development phase, microglia cells move to occupy the inner and outer plexiform layers where they adopt a quiescent phenotype characterized by very small somata and extensively ramified filopodia-like processes (4). They form a mosaic network of evenly distributed non-overlapping cells that provide immunological surveillance to the entire retina by the continuous movement of their processes (4, 34). It is also possible that the dynamic activity of the filopodial structures serves a housekeeping function, enabling microglia eliminate accumulated waste metabolic products and cellular debris in the retinal microenvironment (35). In addition, the extensive processes facilitate close relationships with other retinal cells such as the neurons, promoting the maintenance of their synaptic structures and neurotransmission (3, 36). Indeed, sustained microglia depletion in the retina has been shown to result in the degeneration of photoreceptor synapses with subsequent progressive decline in the magnitude of electroretinographic responses to light stimuli (36).

Cross-talk between microglia and other retinal cells is enabled by the assortment of cell surface molecules on the microglial cell membrane (4, 37). These surface proteins are key regulators of tissue homeostasis that limit unnecessary microglia activation in the healthy retina (9). CD200-CD200R interaction is an important example that induces a negative inhibitory signal to restrain microglia from tissue damaging activation (38, 39). CD200 (previously known as OX2) is a broadly expressed membrane glycoprotein in ganglion cells, photoreceptors, vascular endothelium and the retinal pigment epithelium (RPE), while its receptor, CD200R, is predominantly expressed in retinal microglia (4, 9, 40). Studies on mice lacking CD200 have reported heightened pro-inflammatory responses in experimental animal models of uveoretinitis and exudative form of age related macular degeneration (38–40). Conversely, enhancement of CD200R signaling ameliorates pathological outcome in optic nerve injury and experimental autoimmune uveoretinitis animal models, suggesting that CD200-CD200R interaction could be an exploitable avenue for therapeutic intervention (39, 41).

Sialic acids, covalently linked to cellular membrane proteins and lipids, also contribute to the inhibition of the innate immune system in the CNS including the retina (42, 43). The interaction between polysialic acid chains (PSA) present on the glycocalyx of healthy neurons and Sialic acid binding immunoglobulin-like lectin 11 (Siglec11) receptor of microglia restricts unwanted microglial activation in the brain and retina (42, 44). The inhibitory signals are propagated via immunoreceptor tyrosine-based inhibitory motifs (ITIM) contained in the cytosolic domain of Siglec-11 in microglia (43). Therefore, given its immunomodulatory capacity, several studies have reported Siglec-11-ITIM mediated neuroprotective effects which will be discussed further later in this review (42, 44, 45).

A further neuroimmune regulator is CX3CL1 (fractalkine), a constitutively expressed neuron derived chemokine which binds to its sole receptor CX3CR1 present in microglia and maintains them in a quiescent mode (46). Indeed, numerous studies have demonstrated the immunomodulatory and neuroprotective effects of CX3CL1-CX3CR1 interaction in the brain and retina (46, 47). For instance, transplantation of mesenchymal stem cells engineered to secrete CX3CR1 in the subretinal space inhibited microglial activation and expression of pro-inflammatory factors in light-induced retinal degeneration in rats (48). In contrast, deletion of CX3CR1 in an rd10 retinal degeneration mouse model augmented microglial activation and infiltration into the photoreceptor layers with concomitant increase in photoreceptor demise (49). Remarkably, delivery of exogenous CX3CL1 to the rd10 mouse eye significantly slowed down the tempo of photoreceptor demise, underscoring CX3CL1-CX3CR1 signaling axis as an influential regulator of microglial activity (49).

**Figure 1:**
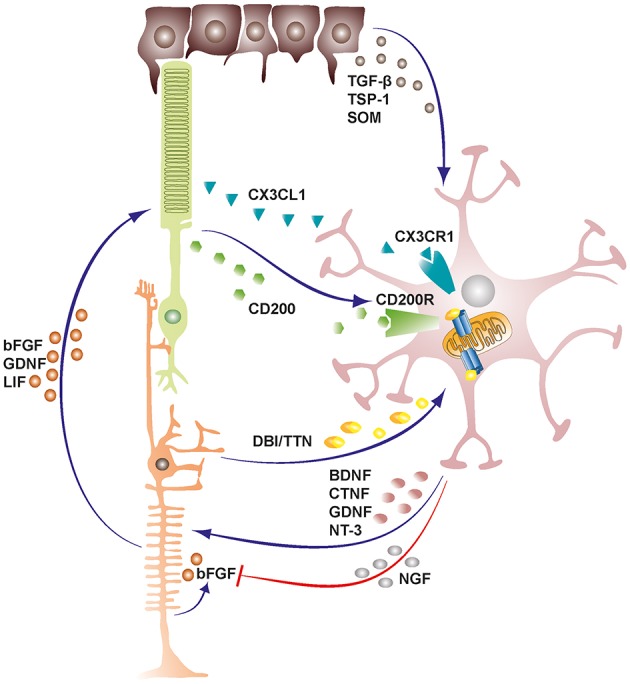
Schematic representation depicting cellular cross-talk between retinal cells and microglia. Retinal cells constantly communicate with microglia via various soluble factors and receptors to restrain microglia from tissue damaging activation and maintain them in a quiescent protective state. The bidirectional communicational between microglia and Müller cells can act as a mediator of neuron-microglia crosstalk. Microglia derived factors may either induce or inhibit release of secondary factors from Müller cells. TGF-β, transforming growth factor beta; TSP-1, thrombospondin-1; SOM, somatostatin; DBI, diazepine binding inhibitor; TTN, triakontatetraneuropeptide; BDNF, brain derived neurotrophic factor; CTNF, ciliary neurotrophic factor; GDNF, glial cell line-derived neurotrophic factor; NT-3, neurotrophin-3; NGF, nerve growth factor; bFGF, basic fibroblast growth factor; LIF, leukemia inhibitory factor.

Microglia also exchange functionally significant signals with Müller cells both in health and disease states, and this bidirectional communication can act as mediator of neuron-microglia crosstalk [Figure 1; (50, 51)]. Indeed, previous studies had demonstrated that certain microglia-derived neurotrophic factors such as BDNF and CNTF were neuroprotective for photoreceptor cells despite these cells not expressing their receptors (52–55). Subsequent studies later established that microglia derived neurotrophic factors interact with Müller cells and induce or inhibit the release of secondary factors including basic fibroblast growth factor (bFGF), leukemia inhibitory factor (LIF) and glial cell line-derived neurotrophic factor (GDNF) that could act directly on photoreceptors and mediate survival or apoptosis during stress conditions in the retina (56–60). Recent investigations have also revealed additional microglia-Müller cell cross-talk via translocator protein (TSPO; 18 kDa) signaling axis, where Müller cells release TSPO's endogenous ligand, diazepam binding inhibitor (DBI) protein, which binds microglial TSPO and suppresses microglial activation during retinal pathology (61).

As a result of the heterocellular signaling and crosstalk in the retina, microglia cells are rendered extremely sensitive to slight changes in their environment, demanding efficient inhibitory mechanisms to maintain them in a quiescent neuroprotective state (4). RPE cells prominently support the maintenance of an immunosuppressive intraocular environment by secreting inhibitory factors into the subretinal space between the neural retina-RPE interface (62). This blocks the undesirable infiltration of microglia cells and other mononuclear phagocytes into the photoreceptor layers and subretinal space, rendering these regions devoid of immune cells in the healthy retina (10, 63). Conversely, in both wet and dry forms of age related macular degeneration, there is an accumulation of mononuclear phagocytes in the subretinal space where they may contribute to disease pathogenesis (63). Some of the inhibitory factors released by RPE cells into the subretinal space include transforming growth factor-β (TGF-β), thrombospondin-1 (TSP-1) and somatostatin (SOM) (64–66).

## Microglia in the Diseased Retina

A sustained chronic pro-inflammatory environment is an important common denominator of retinal degenerative diseases and neurological disorders that affect vision (10). Neuroinflammatory responses in the retina are orchestrated by microglial cells which constitute the resident immune cell population (67). Under acute conditions, microglia-mediated neuroinflammation promotes neuroprotection and regenerative processes and facilitates a rapid return to tissue homeostasis (68, 69). However, under chronic conditions where the insult persists over time, such as in retinal degenerative disorders, microglia become pathologically activated and release exaggerated amounts of inflammatory mediators that promote tissue damage and disease exacerbation (16, 68, 70). Consequently, modulation of microglial reactivity has emerged over the years as a promising therapeutic approach to attenuate neuronal demise and potentially slow down the onset and progression of retinal degenerative diseases. Therefore, the next sections will discuss the role microglia-related mechanisms in major retinal degenerative diseases like age-related macular degeneration, hereditary retinopathies, glaucoma, diabetic retinopathy as well as in aging. In addition, promising regimens that have shown potent immunomodulatory effects in animal models of retinal diseases and/or in human tissue culture as well as patient studies will be discussed.

### Microglia in Age Related Macular Degeneration

AMD, with a global prevalence of ~170 million people, refers to a progressive deterioration of the macula (71). It is a neurodegenerative disease with a multifactorial etiology and is currently considered the leading cause of debilitating vision loss among the elderly (72). Early clinical features of the disease includes fundoscopic manifestation of drusen between the RPE and Bruch's Membrane and fundus autofluorescence that derives primarily from accumulation of lipofuscin granules in the RPE (73, 74). Unfortunately, 15% of patients presenting with early AMD symptoms will advance to the late stages of the disease which progresses in two main forms; (I) Neovascular or “wet” AMD characterized by the ingrowth of immature leaky choroidal blood vessel through the RPE and into the avascular outer retina and (II) Geographic atrophy or “dry” AMD characterized by deterioration of the RPE and associated support functions around the macula with concomitant loss of the overlying photoreceptors (75, 76). While effective treatments involving the inhibition of vascular endothelial growth factor (VEGF) exist for wet AMD, no truly effective form of treatment is available for dry AMD form which affects the majority of patients (77).

There is substantial evidence to indicate the involvement of inflammation and dysregulated innate immunity in the pathogenesis of AMD (73, 75, 78). Dysregulated innate immunity in AMD is associated with complement factors, inflammasome activation, and reactive microglia (71). Wet AMD patients for instance show elevated levels of complement fragments C3a and Ba as well as a wide range of cytokines in their aqueous humor (73, 79). Similarly, soft drusen from AMD donors contain bioactive fragments of complement components C3a and C5a which can induce VEGF expression and predispose patients to choroidal neovascularization (CNV) (80). Drusen from AMD donors can induce inflammasome activation in myeloid and mononuclear cells, suggesting that macular drusen in AMD is a potent pro-inflammatory stimulus (81). Indeed, the widespread accumulation of drusen components as seen in AMD is a prominent chemoattractant stimulus for microglia cells which results in their translocation to the subretinal space (82–84).

Consistently, enlarged amoeboid microglia have been found in the vicinity of RPE cells overlying drusen in retinal sections from dry AMD patients (70). In the outer retina, reactive microglia or microglia-derived factors can induce NLRP3 inflammasome activation in RPE cells with concomitant secretion of the proinflammatory cytokine IL-1β (85–87). NLRP3 inflammasome activation can subsequently induce RPE degeneration via caspase-1-mediated pyroptosis, contributing to AMD pathology (88). Notably, human ocular tissue sections from geographic atrophy or neovascular AMD donors exhibit NLRP3 inflammasome activation (88). Eventually, RPE degeneration causes secondary photoreceptor cell death, resulting in loss of visual function (75). Moreover, the pro-inflammatory environment fostered by accumulating subretinal microglia can directly induce death of nearby photoreceptors (89). We have indeed demonstrated on several instances that conditioned medium from reactive human and murine microglial cells triggers caspase-mediated photoreceptor cell death (85, 90). These findings therefore strongly suggest that microglia reactivity is a driving force in photoreceptor degeneration and progression of retinal degenerative disorders.

Indeed, the role of microglial reactivity in severity of disease has been clearly demonstrated in several mouse models of AMD. However, it is important to note that conclusions drawn from these studies are limited in nature since rodents lack an anatomical macula. Nonetheless, these models effectively mimic distinct features of human pathology including accumulation of immune cells such as macrophages or microglia in the subretinal space, photoreceptor degeneration, choroidal neovascularization, funduscopically visible drusen-like lesions, and RPE degeneration (91). We will therefore discuss a few of these models here to highlight the crucial role played by microglia in AMD pathogenesis. However, for a more detailed discussion on the animal models of AMD, the reader is directed to other articles (91–93).

Bright white light for example can be used to mimic the photoreceptor cell death and retinal degeneration witnessed in human AMD patients (94). Intense light causes bleaching of rhodopsin and excessive phototransduction signaling, resulting in photoreceptor apoptosis in an AP-1 dependent manner (94, 95). This is followed by a robust migration of microglia to the outer retina, making the light-damage model useful for studying physiological as well as pathological consequences of reactive microglia accumulation in the subretinal space as seen in human AMD patients (70). Of note is that microglial activation states and morphological changes are early events that precede recruitment to the outer retina following light induced damage (96). This recruitment of microglia to the outer retina following light damage is mediated in-part by chemokines (C-C motif) ligand 2 (Ccl2) (97) and (C-X3-C motif) ligand 1 (Cx3cl1/ fractalkine) (98) and by the complement anaphylatoxin receptor C5aR (99). Once in the subretinal space, microglia and other mononuclear phagocytes enhance their phagocytic capacity and produce high levels of pro-inflammatory factors including IL-1β, TNF-α, IL-6, CCL2, and iNOS (100). An overt consequence of this massive recruitment of amoeboid microglia in the subretinal space is a severe thinning of the outer nuclear layer as a result of photoreceptor degeneration via pro-inflammatory and phagocytosis mechanisms (16, 61, 89).

Another useful model for dry AMD is generated by immunizing mice with carboxyethylpyrrole (CEP)-adducted proteins (101). CEP is an oxidation product of docosahexaenoic acid (DHA) and is present in the eyes of AMD patients and in their serum at higher levels than in age-matched non-AMD controls (91, 101). This model mimics many aspects of the dry AMD pathology observed in human patients, including presence of sub-RPE drusen like deposits, RPE degeneration and infiltration of phagcoytes around hypertrophied photoreceptors and degenerating RPE (102). However in this model, RPE atrophy is more pronounced in regions devoid of immune cells in their vicinity, suggesting that subretinal mononuclear phagocytes are not involved in initiating RPE pathology, but may rather exert beneficial effects by removing debris released from apoptotic corpses of RPE cells (102). Indeed, some of the infiltrating macrophages found in the subretinal space contained melanin pigment, confirming RPE engulfment (102). This model could therefore be useful in understanding what processes or factors tips the scales toward neurotoxic damaging phagocytes in subretinal microglia.

To study microglial immune responses in wet AMD, a laser induced bruch's membrane photocoagulation model is used (71). Briefly, laser photocoagulation results in the rupture of Bruch's membrane, leading to the ingrowth of immature leaky blood vessels into the outer avascular retina (103). A local inflammatory reaction is triggered at the laser burn site and is accompanied by a rapid recruitment and accumulation of amoeboid microglia and other mononuclear phagocytes (104–106). The recruited mononuclear phagocytes produce high levels of pro-inflammatory cytokines and the pro-angiogenic factor VEGF, worsening disease progression and severity (104, 105). Indeed, blocking the VEGF receptor signaling significantly inhibits microglia accumulation in the laser spots with concomitant reduction in CNV (107). Conversely, blocking ifnar1/IFN-β or CX3CR1 signaling drastically enhances microglia reactivity and recruitment to the laser lesion site, exacerbating the development of CNV lesions in mice (104, 105).

### Microglia in Hereditary Retinopathies

Hereditary degenerations of the human retina are a diverse group of clinically and genetically heterogeneous blinding diseases with more than 260 causal genes identified to date (108). Inherited retinopathies are mostly monogenic, with the causal mutations predominantly occurring in genes expressed in photoreceptors and RPE (4, 109). The most prevalent and severe form of inherited retinopathies is retinitis pigmentosa (RP), where vision loss is brought about by primary degeneration of rods, followed by the degeneration of cones (110). Microglia in human RP patients become reactive in response to signals from degenerating rods and migrate to the photoreceptor layers (70). These bloated microglia in the outer retina of RP patients engage in the phagocytosis of rod debris, as evidenced by their rhodopsin intracellular inclusions (70). In addition to phagocytosis of degenerate cells (70), proposed these reactive microglia in the outer retina exacerbate photoreceptor cell death including adjacent healthy cones by secreting pro-inflammatory neurotoxic factors. Using animal models that recapitulate aspects of human disease, scientists have proven this hypothesis and demonstrated accumulation of reactive microglia in the degenerating retinas that produce high levels pro-inflammatory cytokines and chemokines aggravating photoreceptor demise.

The rodless mouse, discovered approximately 93 years ago by the medical geneticist Keeler, was the first ever reported murine model of retinal degeneration (111). This natural mouse line carries a nonsense mutation in the *Pde6b* gene that codes for the β-subunit of cGMP phosphodiesterase (PDE), and mimics to a great extent the RP in human patients bearing mutations in the human homolog of the gene (112). Rd1 mice display an early onset and a rapid rate of photoreceptor degeneration, such that by 4 weeks of age, only a single layer of photoreceptors consisting predominantly of cone photoreceptors is left in the outer nuclear layer (113). Microglia cells are present in the outer nuclear layer of the photoreceptors by post-natal day 10 where they exhibit a highly proliferative capacity (114). Of note is that their distribution coincides with the spatiotemporal pattern of photoreceptor cell death, implying that microglial cells are intimately engaged with the degenerative process and are not mere bystanders of disease (114). During their migration to the outer nuclear layer of the photoreceptors, microglia lose their filopodial-like processes, and by the time they emerge in the subretinal space, they are fully transformed into amoeboid phagocytes whose pseudopodia can often be seen (114). These bloated infiltrating microglia have been shown to secrete high levels of TNF-α, chemokines CCL2 (alias MCP-1) and (CCL5, alias RANTES) accentuating the ongoing photoreceptor demise (115).

In the retinal degeneration 10 (*rd10*) mouse which harbors a missense mutation in the Pde6b gene but displays a much slower onset of retinal degeneration, a similar infiltration of microglia into the outer nuclear layer is observed at around P21 as the number of apoptotic rods increase (16). Interestingly, microglia activation in *rd10* mice, indicated by the upregulation of microglia specific genes Cx3cr1, Aif1, Irf8, C1qc is already apparent at P12 before the onset of neurodegeneration (116). Upon activation, microglia migrate toward the degenerating photoreceptors and transition into amoeboid cells containing multiple phagosomes where they produce large amounts of the pro-inflammatory cytokine IL-1β and potentiate photoreceptor apoptosis (16). In addition, the reactive outer retina microglia engage in indiscriminate phagocytosis of stressed but living photoreceptors, aggravating neuronal demise, and disease severity (16). Of note is that the number of infiltrating microglia is significantly reduced in *ccr2*^−/−^ *rd10* mice, indicating that the *ccr2/ccl2* signaling pathway plays a crucial role in mobilizing mononuclear phagocytes into the degenerating photoreceptor layer (117). Moreover, the reduced microglial infiltration was also associated with an increase in retinal thickness and function, strongly affirming microglia's involvement in inducing degenerative changes during retinal pathology (117).

In addition to the commonly used *rd1* and *rd10* mutants, *rd7* and *rd8* mutant mouse strains also display widespread microglia activation (118). In the *rd7* mice where retinal degeneration occurs as a result of a spontaneous mutation in the Nr2e3 gene (119), reactive microglia are found under abnormal foldings (retinal rosettes) that develop in the outer nuclear layer and inner segments of photoreceptors (118). These reactive microglia phagocytose large amounts of debris between photoreceptor and RPE layers, resulting in the accumulation of lysosomes containing autofluorescent material (118). Activated-lysosome-laden microglia, which appear as autofluorescent dots in fundus images, secrete high amounts of pro-inflammatory cytokines IL-1β, IL-6, and TNF-α thereby accelerating retinal degeneration (118). Similarly, in the *rd8* mice bearing a mutation in the Crb1 gene, increased amounts of microglia/macrophages positive for pro-inflammatory markers CD16, MHC-II, and iNOS accumulate in the subretinal space when compared to age matched wild type controls (120). This enhanced accumulation of reactive microglia overexpressing complement (C3 and CFB) and pro-inflammatory (TNF-α and NFκB) genes, is a specific response to degenerative changes that occur in the *rd8* retina including development of retinal dystrophic lesions (120, 121).

### Microglia in Diabetic Retinopathies

Diabetic retinopathy (DR) is a severe ocular complication of diabetes mellitus and is also the leading cause of blindness among the working age populations of industrialized regions (122). DR can be clinically divided into two forms; (i) an early non-proliferative form (NPDR) characterized by increased vascular permeability, retinal microvasculature degeneration, basement membrane thickening, and loss of pericytes in the retinal capillaries (ii) an advanced proliferative form (PDR) involving pathological neovascularization, vitreous hemorrhage, and retinal scars and detachment (123). Based on vascular abnormalities such as acellular capillaries, microscopic capillary loss, and microaneurysms, DR has traditionally been regarded as a classical microvascular disease (122). However, in recent years, numerous studies have highlighted the crucial role played by inflammation in the pathogenesis of DR (124–126).

Early indications of inflammation involvement in the pathogenesis of DR came from a study reporting a low incidence and high regression rate of DR when diabetic patients were treated with salicylates for rheumatoid arthritis complications (127). Since then, ample evidence exists to show that DR is a low grade chronic inflammatory condition characterized by leukostasis, RPE, and endothelial cell damage and associated blood retinal barrier (BRB) alteration. Subsequently, these events compromise the immune suppressive environment in the retina resulting in increased expression of pro-inflammatory factors (124, 128, 129). Indeed, several clinical studies have shown that the levels of many pro-inflammatory cytokines including TNF-α, IL-1β, IL-6, and IL-8 are elevated in the vitreous of DR patients (124, 128). In addition, persistent hyperglycaemia results in increased polyol and hexosamine pathways flux, inducing cellular oxidative stress and the generation of advanced glycation or lipoxidation end products (AGEs or ALEs) (122, 130). High levels of pro-inflammatory cytokines and the accumulation of AGEs and ALEs compromise cellular physiology and induce microglia activation (131, 132). Furthermore, oxidative stress induced by the noxious hyperglycaemic environment can trigger NFκB-mediated inflammatory responses in retinal microglia (133).

In human DR, hypertrophic and amoeboid microglia are present at different stages of the disease (134). In the NPDR form, there is a moderate increase in the number of reactive microglia which are mostly clustered around perivascular vessels and fresh hemorrhages in microaneurysms (135). In the intermediate pre-proliferative form, there is a dramatic increase in reactive microglia which cluster around cotton-wool spots and dilated vessels (135). Lastly in the PDR form, a marked increase in reactive microglia number is seen in the ganglion cell layer and around new immature blood vessels in the nerve fiber layer and the optic nerve head where the proliferative process is most prominent (135). Strikingly, reactive microglia in human DR are closely associated with perivascular compartments where they are postulated to exacerbate vascular permeability by propagating inflammatory responses (135, 136).

Similarly in murine models, changes to retinal microglia are a prominent feature of diabetic retinopathy (137, 138). In the Ins2^Akita^ mouse model which harbors a spontaneous missense mutation in the insulin 2 gene, discrete pockets containing bloated microglia with shorter less branched processes can be observed in the diseased retina (138). Moreover, the normal laminar arrangement of the retinal microglia is markedly disrupted in these mice from 10 weeks of age (137). These DR induced microglia morphological alterations might result partly from disruptions in microglial Cx3cr1 signaling, as the detrimental morphological abnormalities are exacerbated in the absence of Cx3cr1 signaling (137).

In the Goto-Kakizaki (GK) inbred rat model of type II diabetes, numerous Iba1^+^ amoeboid microglia/macrophages overexpressing inducible form of nitric oxide synthase (iNOS) can be found all over the retina and in the subretinal space following 12 months of hyperglycaemia (139). The enhanced trafficking of microglia/macrophages to the subretinal space at week 12 is correlated with a reduction in the number of “tunnel-like” invaginations which mediate a physiological transcellular pathway in RPE cells (139). Alteration of this transcellular pathway and subsequent subretinal accumulation of activated microglia/macrophages induces morphological abnormalities in the outer retina including disorganization of photoreceptor outer segments (139). Notably, intraocular injection of a protein kinase C zeta (PKCζ) inhibitor suppresses iNOS expression in microglia/macrophages and impairs their recruitment to the subretinal space (139).

Microglia reaction is also elicited in pharmacologically induced diabetes where toxic glucose analogs alloxan and streptozotocin (STZ) are used to preferentially destroy pancreatic beta cells and induce a state of insulin-dependent diabetes (134, 140, 141). Starting at 4 months of age, an upsurge of microglia is observed in the different retinal layers and especially the outer plexiform and ganglion cell layer in STZ treated rats (134). There is also a significant shift in microglia phenotype toward activated amoeboid cells with hypertrophied cell bodies (134, 142). As the disease progresses, microglia in the outer plexiform layer extend their processes into the outer nuclear layer, with some CD11b positive microglia occasionally being found in the photoreceptor layers and subretinal space (134). Interestingly in alloxan treated mice, microglia morphological changes including shortened processes and hypertrophied cellular somas precede neuronal apoptosis and BRB breakdown in this model, implying that microglia cells play an important role in the onset and development of DR (141).

### Microglia in Glaucoma

Glaucoma refers to a distinctive group of optic neuropathies characterized by the progressive demise of retinal ganglion cells (RGCs) and their axons, thinning of the retinal nerve fiber layer and cupping of the optic disc (143). Glaucoma, estimated to affect ~79.6 million people by 2020, is the most frequent cause of irreversible blindness worldwide (144, 145). Major risk factors for the development and progression of glaucoma include old age, race, ocular and systemic hypertension, diabetes, and high myopia (146–148). To date, the only proven therapeutic approach to prevent development and slow down disease progression is the lowering of intraocular pressure (IOP) via drug treatment, laser therapy or surgery (51, 143). However, despite effectiveness of the intraocular pressure-lowering approach which eliminates the primary source of injury, evidence exists of a secondary degeneration that persists, affecting neighboring neurons and continuing the pathological process (149–151). An important mechanism that has been proposed to provoke such secondary degeneration of the RGCs in optic nerve disorders is the dysregulation of innate immunity and the associated neurodegenerative inflammatory responses (152).

Consistent with this hypothesis, aqueous humor from glaucoma patients have been shown in several studies to contain higher levels of pro-inflammatory cytokines and chemokines, suggesting that neuroinflammatory processes play a key role in glaucomatous neurodegeneration (153–156). Microglia, the resident tissue macrophages, are the principal sources of pro-inflammatory mediators in the retina and have been shown in both human and experimentally induced glaucoma in rodents to be central players in perpetuating the neuroinflammatory process in glaucoma (4, 157–159). In human glaucoma eyes, clusters of large amoeboid, reactive microglia gather around the compressed lamina cribrosa and its surrounding blood vessels, forming concentric circles (159). Enlarged reactive microglia, occurring either singly or in clusters, are also found in the parapapillary chorioretinal region (where the RPE and the bruch's membrane terminate) of glaucomatous optic nerve heads (ONH) (159). Proteomic and subsequent immunohistochemical analysis detected abundant expression of toll like receptor 2 (TLR2), TLR4, and TLR7 in microglia of diseased patients, implicating TLR signaling as a pathomechanism in glaucomatous neurodegeneration (160). Moreover, these reactive microglia were shown to express abundant levels of matrix metalloproteinases (MMP) 1, MMP-2, MMP-3, and MMP-14, pro-inflammatory factors TNF-α and NOS-2 and the anti-inflammatory cytokine TGF-β (158). The high expression of TGF-β suggests that microglia attempt to downregulate the degenerative reactions in the glaucomatous ONH, but as the disease progresses, their contribution turns detrimental leading to neurodegeneration and degradation of the extracellular matrix (158).

Murine models of glaucoma where increased ocular hypertension occurs naturally or is experimentally induced using genetic manipulation or surgical procedures have been instrumental in understanding microglia's role in disease pathogenesis (161). Using DBA/2J (D2) mice, an established model of chronic inherited glaucoma carrying spontaneous mutations in TYRP1 (tyrosinase-related protein 1) and GPNMB (glycosylated protein nmb), microglia activation and proliferation was shown to occur at stages prior to overt RGCs neurodegeneration (162, 163). These findings provide evidence supporting an active involvement of microglial activation in the onset and progression of RGCs neurodegeneration in the glaucomatous retina (162, 163). Microglia mediate the neurodegeneration of RGCs, at least in-part, by secreting high amounts of TNF-α (164). Consistently, mice deficient for TNF-R1 are significantly protected against RGCs loss following optic nerve crush when compared to control animals with a functional TNF death receptor signaling (164). Moreover, both TNF-α and its receptor TNF-R1 are upregulated in glaucomatous retinas in the RGCs and retinal glial cells, respectively, suggesting a direct contribution of the TNF-α signaling cascade in optic nerve degeneration in glaucoma (165, 166). Notably, blocking TNF-α activity with Etanercept, a clinically approved agent, inhibits microglial inflammation with concomitant attenuation of axonal degeneration in the optic nerve and RGC demise (167).

Nitric oxide (NO) synthesized by microglia also plays a role in the pathophysiology of glaucoma (168, 169). Increased levels of iNOS and NO have been shown to occur in the ONH of glaucomatous patients and experimental animal models of glaucoma (168–171). Importantly, pharmacological inhibition of NO synthesis using timolol and aminoguanidine in rats with elevated IOP drastically suppresses the degeneration of RGCs, showing that NO-mediated mechanisms contribute to neuronal cell death during glaucoma (169, 172).

Increased IL-6 expression has also been reported as a key component of the retinal microglia response in glaucoma, although the available evidence is contradictory (173–175). Early *in-vitro* experiments conducted on primary glia cultures isolated from rat retina demonstrated that microglia, and not astrocytes were the primary sources of IL-6 following elevated pressure conditions (175). Interestingly, microglial secreted IL-6 was shown to significantly abate apoptotic cell death in RGCs injured by elevated pressure, suggesting a beneficial effect of this cytokine in countering proapoptotic signals in the glaucomatous retina (174). In contrast, other investigations have reported that while an IL6 deficiency promotes an anti-inflammatory, pro-survival retinal environment, it also induces an exaggerated TNF-α response in the presence of glaucomatous stressors such as ocular hypertension (176). Moreover, *IL6*^−/−^ mice are protected from IOP induced structural degeneration of the optic nerve and subsequent decrease in vision acuity, suggesting that IL-6 may play a specific role in the progression of RGC axonopathy and vision loss (173). Further studies are therefore needed to delineate ambiguities and lack of clarity in reported outcomes prior to the development of IL-6 as a therapeutic target.

## Targeting Microglia for the Treatment of Retinal Degenerative Diseases

Microglia participates in both physiological and pathophysiological functions in the retina (4). Therefore, despite the fact that neuroinflammatory responses from overly reactive microglia play a critical role in the onset and progression of retinal degenerative disorders, complete blocking of retinal microglial functions would result to undesirable effects (36). Hence, valid immunotherapeutic approaches for the treatment of retinal degeneration should be those that inhibit dysregulated microglial-mediated pro-inflammatory responses and/or simultaneously enhance their beneficial neuroprotective functions. In the remainder of this review, we discuss promising therapeutic strategies that have been used to modulate microgliosis and improve disease outcome during retinal pathologies.

### Polysialic Acid Receptors

**Figure 2:**
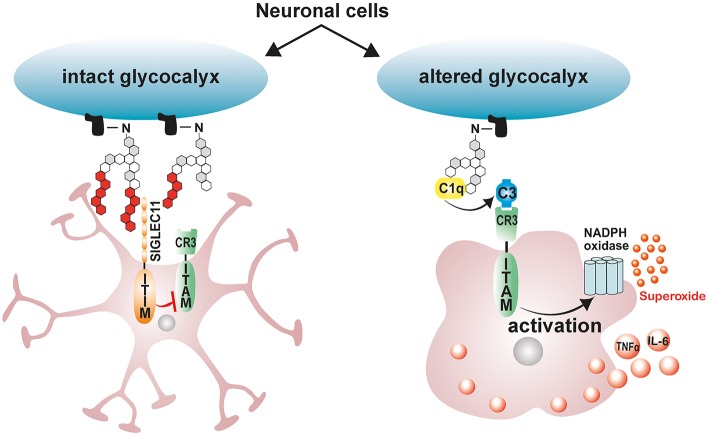
Schematic model depicting the immunomodulatory effects of neuronal polysialic acids on microglia. Healthy neuronal cells have an intact glycocalyx displaying a polysialic acid (PolySia) cap. The PolySia cap is recognized and bound by Siglec-11, an inhibitory ITIM-signaling receptor. This interaction between neuronal PolySia and Siglec-11 inhibits microglia activation and maintains retinal homeostasis. However, during pathological conditions, neuronal glycocalyx is altered, leading to degradation of sialic acid caps. The exposed neuronal glycocalyx is then opsonized by complement component C1q, leading to subsequent recognition by ITAM-containing complement receptor 3 (CR3) in microglia and activation of inflammatory signaling and ROS production. ITIM, immunoreceptor tyrosine-based inhibition motif; ITAM, immunoreceptor tyrosine-based activation motif.

In the vertebrate nervous system, healthy neuronal cells carry a polysialic acid (PolySia) cap on their glycocalyx made up of α2-8-glycosidically linked N-acetylneuraminic acid residues and which are attached by means of Hildebrandt et al. (177) and Schnaar et al. (178). The glycosylation state of healthy neurons is vigilantly monitored by cell surface carbohydrate binding receptors on microglia referred to sialic acid-binding immunoglobulin-like lectins (Siglecs) (43). Neural cell-adhesion molecule (NCAM), involved in cell–cell interactions and cell–extracellular-matrix adhesion, is the most prominent neuronal protein modified by PolySia (179). Polysialylated neuronal cell adhesion molecule (PolySia-NCAM) binds a CD33-related, primate lineage-specific Siglec-11 receptor on microglia, and this interaction serves to limit microglia immune responses and maintaining them in a quiescent neuroprotective state [Figure 2; (43)]. The inhibitory signaling of Siglecs is mediated through conserved ITIM-domains contained in their cytosolic tails (180). Upon ligand binding, ITIM is phosphorylated by Src-kinases, leading to a subsequent recruitment of protein-tyrosine phosphatases Src-homology region 2 domain-containing phosphatase 1 (SHP1) and SHP2 (180). Once recruited, SHPs dephosphorylate activated tyrosine residues on immunoreceptor tyrosine-based activation motif (ITAM), inhibiting Syk kinase mediated inflammatory signaling, ROS production and phagocytosis (180–183). Indeed, the interaction of ectopically expressed human Siglec-11 in mouse microglia with PolySia residues on neurons potently downregulates LPS-induced pro-inflammatory gene transcription and phagocytic capacity in microglia, confirming that ITIM-signaling impedes the inflammatory, and phagocytosis-associated ITAM-Syk cascade (181).

Under pathological conditions such as elevated inflammation and oxidative stress, degradation of sialic acid caps occurs leaving neurons and other cells with a damaged glycocalyx (184–186). During such pathological conditions, soluble sialic acid residues actually accumulate in serum signifying the removal of sialic acid caps from glycoproteins (184, 185). Desialylated neuronal glycocalyx are then opsonized by complement component C1q, leading to their subsequent phagocytosis and clearance by the activating-signaling receptor complement receptor 3 (CR3) in microglia/macrophages (43). Consistently, blockage of CD11b as part of the complement receptor 3 (CR3) attenuates desialylated neurite phagocytosis by macrophages (187). However, excessive C1q mediated microglial phagocytosis of desialylated neurons would exacerbate ongoing neuronal loss in retinal degenerative disorders (188). This phenomenon is clearly observed in glaucoma mouse models where C1q localizes with neuronal synapses early on in the disease, and blocking neuronal opsonization by depleting C1q protects mice from glaucoma induced neurodegeneration (188). These findings therefore suggest that treatment with exogenous polysialic acids may be neuroprotective by limiting disease induced microglial inflammatory responses and phagocytic capacity.

Indeed, treatment of human macrophages challenged with LPS or amyloid-β_1−42_ with nanomolar concentrations of low molecular weight polySia with an average degree of polymerization 20 (polySia avDP20) significantly reduces pro-inflammatory gene transcription, inflammation-induced phagocytosis, and oxidative burst (44). Similarly in the retina, treatment with polySia avDP20 was shown to significantly reduce microglial activation, vascular leakage and production of deleterious ROS in humanized transgenic mice expressing SIGLEC-11 following laser-induced choroidal neovascularization (42). Interestingly, higher doses of polySia avDP20 were shown to block alternative complement activation and membrane attack complex formation in the diseased retina but in a SIGLEC-11 independent manner (42).

### Translocator Protein Ligands

**Figure 3:**
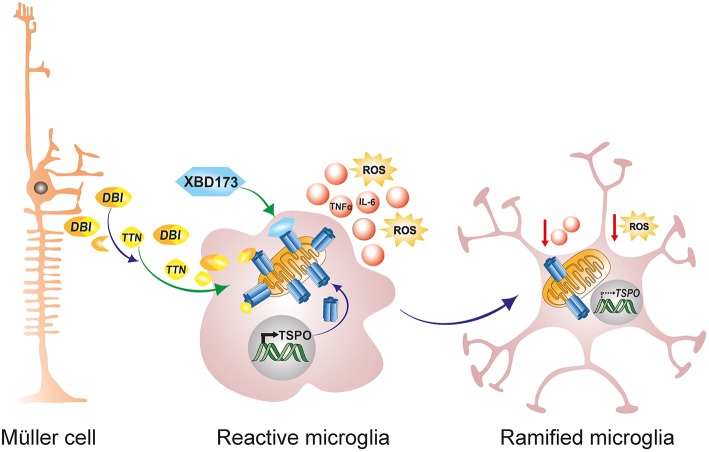
Endogenous and exogenous TSPO ligands dampen microglial activation. During retinal pathophysiology, microglia upregulate TSPO expression while Müller cells simultaneously upregulate the production and secretion of the endogenous TSPO ligand DBI. Secreted DBI can subsequently be cleaved extracellularly into the biologically active product TTN. Extracellular DBI and TTN are taken up by microglia, and their binding to the TSPO receptor serves to limit the magnitude of microglial inflammatory responses. DBI, diazepine binding inhibitor; TTN, triakontatetraneuropeptide; TSPO, translocator protein 18 kDa.

Translocator protein (18 kDa; TSPO), formerly referred to as the peripheral benzodiazepine receptor (PBR), is a highly conserved 5α-helical transmembrane protein located on the outer mitochondrial membrane (OMM) (189). TSPO exhibits a high constitutive expression in steroidogenic tissues such as adrenal glands, gonads and the placenta, but is very weakly expressed in the normal healthy brain (190, 191). However, during neuropathology, there is a strong increase in TSPO protein expression in the brain which colocalizes predominantly with activated microglia (190, 192, 193). This strong upregulation of TSPO in the brain from a very low baseline led to the development of numerous TSPO positron-emission tomography (PET) ligands for the non-invasive imaging of neuroinflammation (194, 195). Similarly, during retinal inflammation and disease, there is a strong upregulation of TSPO in reactive migratory microglia, and this induction accurately marks the extent and duration of retinal inflammation (190, 193). Interestingly, as activated microglia upregulate TSPO expression during retinal inflammation and disease, astrocytes, and Müller cells upregulate the production and secretion of Diazepam binding inhibitor (DBI), a 9 kDa endogenous TSPO protein ligand (61). DBI is then taken up by microglia cells, and the binding of DBI to microglial TSPO serves to limit the magnitude of microglial inflammatory responses [Figure 3; (61)]. Secreted DBI can also be cleaved extracellularly by acidic endopeptidases into its biologically active cleavage product triakontatetraneuropeptide (TTN) (196). TTN interaction with retinal microglial TSPO similarly suppresses inflammatory responses and facilitates a return of activated microglia to baseline quiescence (61, 196). Exploiting this endogenous immunomodulatory pathway, we tested the ability of a synthetic and highly specific TSPO ligand, XBD173 (AC-5216, emapunil), to influence microglial reactivity in the acute white light-induced retinal degeneration mouse model (193). White light exposure is an environmental risk factor that contributes to the faster onset and progression of human retinal degeneration disorders such as AMD and Retinitis Pigmentosa (197–200). Consistently, rodents exposed to bright white light experience a synchronized burst of photoreceptor cell death and thinning of the outer nuclear layer (94, 95, 193). Photoreceptor demise is brought about by rhodopsin bleaching and excessive phototransduction signaling, which creates an intracellular death signal that translocates transcription factor AP-1 to the nucleus to mediate photoreceptor apoptosis (59, 94, 95). Using this model, we demonstrated that the TSPO ligand XBD173 markedly inhibited accumulation of amoeboid microglia in the outer retina with concomitant preservation of the outer nuclear layer (193). Moreover, XBD173 efficiently suppressed pro-inflammatory induced gene expression and morphological transition in microglia with concomitant reduction in microglial neurotoxicity on cultured photoreceptors (190). However, the precise mechanisms involved in the TSPO ligands neuroprotective effects remain largely unknown, but likely involves, at least in part, enhanced steroidogenesis.

Indeed, the most studied physiological function of TSPO involves the translocation of cholesterol from the outer to the inner mitochondrial membrane as a rate limiting step for steroidogenesis (189, 201). TSPO's implication in steroidogenesis has been based upon its localization to the OMM, presence of a high affinity cholesterol recognition amino acid consensus (CRAC) motif, high expression in steroidogenic cells and the ability of TSPO ligands to stimulate steroid synthesis (202–204). In our own *in-vitro* experiments in the lab, we have demonstrated that treatment of microglia cells with XBD173 induces pregnenolone synthesis, and that inhibition of this process with aminoglutethimide partially abolishes XBD173 therapeutic effects (190). Similarly, the endogenous ligand TTN induces synthesis of pregnenolone derived Dehydroepiandrosterone (DHEA), inhibiting microglia-mediated proinflammatory responses (61). Outside the retina, DBI and synthetic TSPO binding ligands stimulates cholesterol translocation to the inner mitochondrial membrane increasing pregnenolone formation by isolated adrenocortical, Leydig and glia cell mitochondria (205, 206). In addition, gene silencing to inhibit DBI expression in Leydig cells was shown to significantly suppress hormone-induced steroidogenesis but not adenylate cyclase nor cholesterol side chain cleavage (P450SCC) enzyme activities (207). Of note is that the absence of TSPO in mutant rats causes increased neutral lipid accumulation in the adrenal glands and testis, reduced circulating testosterone and undetectable levels of the neurosteroid allopregnanolone (208). Together, these findings strongly suggest that TSPO and its ligands regulate steroid and neurosteroid levels.

Steroid hormones have subsequently been shown to attenuate neuroinflammatory reactions via autocrine and paracrine signaling mechanisms (209). Binding of steroids to their cytoplasmic and nuclear bound receptors results in the rapid inhibition of pro-inflammatory gene transcription via both genomic and non-genomic mechanisms (210). In the retina, a synthetic progesterone, norgestrel, exerts potent neuroprotective effects in the rd10 mouse model of human RP by reducing the extent of neuronal apoptosis with concurrent preservation of the photoreceptor layer (211). Norgestrel mediated rescue of stressed photoreceptors was shown to be mediated by the pleiotropic cytokine LIF, the pro-survival growth factor bFGF and the fractalkine-CX3CR1 signaling cascade (211–213). In addition, norgestrel directly targets reactive microglia in the rd10 mouse, attenuating their pro-inflammatory gene expression and NO production and significantly lessening microglial neurotoxicity on photoreceptor like cells *in vitro* (214). Moreover, the steroid hormone 17β-estradiol (βE2), which can be synthesized in the brain from testosterone, has been shown to protect rats against light-induced retinal degeneration by upregulating the NRF2-antioxidant pathway and reducing ROS production (215). In summary, TSPO ligands, working in part via inducing neurosteroidogenesis, present as attractive therapeutic regimens to dampen inflammatory responses from overly reactive microglia during retinal degenerative diseases.

### Interferon-β

Interferon-β (IFN-β) belongs to the type I IFN family which are best known for their ability to induce a cellular antimicrobial state during a viral or bacterial infection (216, 217). IFN-β also possesses strong immunomodulatory properties, making it a first-line immunotherapy against relapsing remitting Multiple Sclerosis (MS) (217). IFN-β mediates neuroprotection against MS, an inflammatory and demyelinating CNS disorder characterized by multifocal brain lesions, by recruiting microglia to the lesion sites, enhancing their phagocytic capacity and accelerating clearance of accumulated myelin debris (218–220). Conversely, experimental autoimmune encephalomyelitic (EAE) mice which mimic the disease process of MS develop an exacerbated disease course accompanied by microglial hyperactivation and increased lethality in the absence of myeloid IFN-β signaling (221). Therefore, based on the immunomodulatory properties of IFN-β in MS neuropathology, we hypothesized that IFN-β may confer neuroprotection against chronic inflammation observed in neovascular age related macular degeneration (AMD) and thereby improve disease outcome.

We used the laser-induced choroidal neovascularization (CNV) mouse model that mimics features of exudative AMD to test this hypothesis (103). Briefly, laser photocoagulation results in the rupture of the Bruch's membrane, leading to the ingrowth of immature leaky blood vessels from the choroid to the subretinal space (103). Of note is that Bruch's membrane rupture is accompanied by rapid recruitment of myeloid cells to the lesion sites with concomitant elevation of proinflammatory and angiogenic factors, triggering the formation and growth of new blood vessels from the choroid (75, 222). Using this model, we demonstrated that IFN-β strongly inhibits microglia/macrophage activation and recruitment and induces a transition of microglia morphology toward a neuroprotective ramified with more cellular processes (104). Moreover, IFN-β treated animals showed considerable improvement in disease outcome signified by a reduction in vascular leakage and neoangiogenesis (104). These results corroborated those of a previous study which showed that local administration of IFN-β exacerbated wound healing of retinal lesions produced by laser photocoagulation in rabbits (223). Conversely, global or microglia specific conditional deletion of IFN-β/IFNAR1 signaling in mice resulted in an exacerbated disease marked by reactive microgliosis and enhanced vascular leakage (104). These findings strongly suggest that Ifnar1/IFN-β signaling, particularly in microglia, prevents chronic inflammation and pathological neovascularization in the retina and could be therapeutically targeted in wet AMD and other retinal inflammatory disorders. Remarkably, IFN-β treatment was reported to completely reverse subfoveal neovascularization and choroiditis in an MS patient, underscoring the importance of IFN-β in the management of ocular inflammation and neovascularization (224).

Several mechanisms have been proposed for IFN-β immunomodulatory and anti-angiogenic effects. One such mechanism involves the IFN-β induced expression of suppressor of cytokine signaling 1 (SOCS1) and SOCS3 (225, 226). IFN-β and other type I IFNs activate the transcription of SOCS1 and SOCS3 as part of a negative feedback loop to curb prolonged signaling by pro-inflammatory cytokines via the JAK-STAT pathway (227). Once produced, SOCS1 and SOCS3 are recruited to cytokine receptors where they inhibit the catalytic activity of JAK tyrosine kinases and negatively regulate inflammatory responses (228, 229). This phenomenon has been clearly demonstrated in the retinas of experimental autoimmune uveoretinitis (EAU) mice lacking SOCS3 in myeloid cells (230). These retinas showed elevated levels of pro-angiogenic factor VEGF-A and pro-inflammatory cytokines IL-1β, TNF-α, and IFN-γ (230). Consequently, myeloid-specific SOCS3 deficient mice displayed exaggerated retinal degeneration and accelerated retinal angiogenesis compared to their wildtype counterparts (230). In contrast, overexpression of SOCS1 was shown to protect retinal cells against staurosporine and H_2_O_2_-induced apoptosis (231). Notably, the overexpression SOCS1 in EAU mice significantly lessens disease severity by suppressing inflammatory chemokine expression and inhibiting recruitment of inflammatory cells into the retina (231).

IFN-β is also known to mediate its immunomodulatory effects via the noncanonical activation of the PI3K–AKT–mTOR pathway (232, 233). A recent study indeed revealed a central role of IFN-β signaling in the regulation of the PI3K–AKT pathway, by demonstrating that neurons from *Ifnb*^−/−^ mice exhibited striking reductions in Pi3K and Akt mRNA and protein levels (234). Moreover, phosphorylated Pi3k and Akt, which signify active signaling, were even more pronouncedly reduced in the IFN-β incompetent neurons (234). Following activation, PI3K–AKT–mTOR pathway has been shown to exert anti-inflammatory and neuroprotective effects (215, 235). Activation of mTORC1, downstream of PI3K–AKT induces the phosphorylation of STAT3 at Ser^727^, thereby enhancing expression of the counter regulator of inflammatory signaling, SOCS3 (236). Of note, the pharmacological blockade of Pi3k-Akt pathway abolishes the antioxidative and neuroprotective effects conferred by the steroid hormone βE2 following light induced retinal degeneration in rats (215). Similarly, in LPS activated primary rat microglia, pharmacological blockade of the Pi3k-Akt-mTOR pathway significantly enhances inflammatory cyclooxygenase-2 (COX-2) activity and elevates production of associated downstream prostanoids PGE_2_ and PGD_2_ (237, 238).

**Figure 4:**
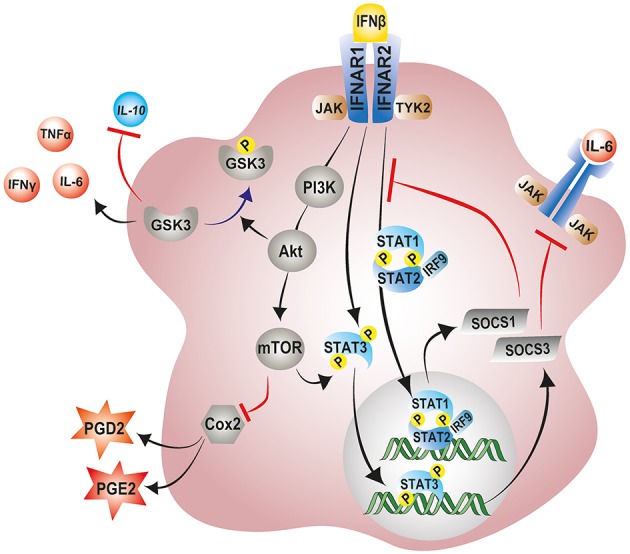
Modulation of microglial inflammatory responses by IFN-β signaling. IFN-β ligation to its receptor complex, IFNAR, triggers activation of the associated tyrosine kinases JAK1 and TYK2 and the subsequent phosphorylation of STAT1, STAT2, and STAT3 transcription factors. Phosphorylated STAT1 and STAT2 can recruit IRF-9 to the form the trimolecular complex STAT1–STAT2–IRF9 (ISGF3). Phosphorylated STAT3, and ISGF3 translocate to the nucleus and induce the transcription of various interferon-stimulated genes including SOCS1 and SOCS3 as part of a negative feedback loop. SOCS1 and SOCS3 inhibit excessive signaling by pro-inflammatory cytokines via the JAK-STAT pathway. IFN-β can also non-canonically activate the PI3K–AKT–mTOR pathway and suppress microglial inflammatory responses. mTORC1, downstream of PI3K–AKT can phosphorylate and activate STAT3, thereby enhancing expression of SOCS3. Activation of AKT downstream of IFN-β-PI3 inhibits GSK-3 activity through phosphorylation, negatively regulating GSK-3 mediated inflammatory responses, and promoting IL-10 expression. JAK1, Janus kinase 1; TYK2, tyrosine kinase 2; STAT, signal transducer and activator of transcription; IRF9, interferon regulatory factor 9; PI3K, phosphoinositide 3-kinase; AKT, protein Kinase B; mTOR, mammalian target of rapamycin; GSK-3, glycogen synthase kinase 3.

Furthermore, IFN-β mediated activation of the PI3-AKT pathway negatively regulates the activity of the pro-inflammatory enzyme glycogen synthase kinase-3 (GSK-3) (239). GSK-3 is a critical mediator of inflammatory responses in the CNS and has been shown to downregulate production of anti-inflammatory cytokine IL-10 and promote that of pro-inflammatory cytokines TNF-α, IFN-γ, and IL-6 in microglia and macrophages (239, 240). Activation of AKT downstream of IFN-β-PI3 causes inhibition of GSK-3 by serine phosphorylation, negatively regulating inflammatory responses in the CNS [Figure 4; (239)]. Notably, treatment of *rd10* mice with a small molecule inhibitor of GSK-3 suppresses pro-inflammatory responses in the retina with concomitant reduction in photoreceptor demise and preservation of visual function (241). However, further research in this area is necessary to delineate the contributions of this pathway to the immunomodulatory effects of IFN-β on microglia during retinal inflammation and disease.

### Minocycline

Minocycline is a highly lipophilic, semi-synthetic tetracycline derivative which is mainly used in the treatment of acne vulgaris and rheumatoid arthritis (242). However, due to its ability to readily penetrate the CNS and its profound anti-inflammatory properties, minocycline has recently emerged as a powerful immunomodulatory drug that is well-suited for CNS disorders (242, 243). Minocycline blocks microglial activation in response to a variety of inflammatory stimuli by inhibiting Toll-like receptor 2 (TLR2) and TLR4 signaling as well as several MAP kinases including p38, c-Jun-N-terminal activated protein kinase (JNK) 1/2, and extracellular signal regulated kinase (ERK) 1/2 (244, 245). In addition, minocycline inhibits NFκB transcriptional activity by suppressing the degradation of inhibitor of kappa B-alpha (IκBα) (244, 246).

Consistent with its anti-inflammatory properties, minocycline benefits during retinal pathophysiology have been confirmed in several experimental models. In a light-induced retinal degeneration mouse model, minocycline treatment was shown to significantly inhibit photoreceptor apoptosis and preserve retinal structure (89). Moreover, minocycline potently inhibited microgliosis and the accumulation of reactive amoeboid microglia in the subretinal space following light induced retinal damage (89). In an *rd10* mouse model of human RP, treatment with minocycline significantly reduced microglia-mediated photoreceptor apoptosis, improving retinal structure and function (247). In a rat model of diabetic retinopathy, minocycline treatment represses the release of pro-inflammatory cytokines IL-1β, and TNF-α with concomitant reduction in caspase-3 mediated apoptosis in the retina (248). Similarly, in a streptozotocin-induced rat model of DR, minocycline treatment inhibited the abnormal expression of poly (ADP-ribose) polymerase 1 (PARP1), a chromatin-associated enzyme that promotes proinflammatory responses in glial cells, with concurrent reduction in the number of apoptotic cells in the diabetic retina (249). In the DBA/2J chronic mouse model of glaucoma, minocycline inhibited microglial activation, and increased the fraction of microglia in a ramified neuroprotective state (250). Of note, minocycline improved RGC axonal transport and integrity in the glaucomatous retina (250).

Minocycline protective effects have also been examined in the human retina (251). Retinitis pigmentosa mediated decline in visual field was shown to be reversed upon long term minocycline treatment (251). In a NIH proof of concept clinical study involving patients with diabetic macula edema (NTC01120899), a vision threatening form of DR, treatment with oral minocycline reduced abnormal vascular permeability and leakage with concomitant improvement in vision acuity (252). Taken together, these findings demonstrate the broad range immunomodulatory and neuroprotective effects of minocycline and underscore the importance of further testing to establish this antibiotic as an immunotherapy against retinal pathologies.

## Conclusion

Studies using rodent models in the past decade have been instrumental in understanding the role of microglial responses in retinal pathologies. Ample evidence generated from these studies shows unequivocally that microglia reactivity and chronic inflammation is a common hallmark of various retinal pathologies, and that pharmacological targeting of overly reactive microglia ameliorates disease pathogenesis. However, why and how immunologic checkpoints become overwhelmed resulting in dysregulation of microglial inflammatory responses remains poorly understood. Therefore, prior to development of microglial-based therapeutic approaches, further studies are needed to understand the etiological factors and molecular mechanisms that propagate microglial responses during retinal degeneration. In addition, owing to the numerous overlapping insults that predispose retinal pathologies, it will be prudent to develop microglia based therapeutic approaches that will complement, or even synergize, other retinal therapeutic approaches.

## Author Contributions

All authors listed have made a substantial, direct and intellectual contribution to the work, and approved it for publication.

### Conflict of Interest Statement

The authors declare that the research was conducted in the absence of any commercial or financial relationships that could be construed as a potential conflict of interest.
